# Influence of gender, sexual orientation, and need on treatment utilization for substance use and mental disorders: Findings from the California Quality of Life Survey

**DOI:** 10.1186/1471-244X-9-52

**Published:** 2009-08-14

**Authors:** Christine E Grella, Lisa Greenwell, Vickie M Mays, Susan D Cochran

**Affiliations:** 1 UCLA Integrated Substance Abuse Programs, Semel Institute for Neuroscience and Human Behavior, Department of Psychiatry and Biobehavioral Sciences, University of California, Los Angeles, USA; 2 Department of Psychology and Department of Health Services, School of Public Health, University of California, Los Angeles, USA; 3 Center for Research, Education, Training and Strategic Communications on Minority Health Disparities, University of California, Los Angeles, USA; 4 Department of Epidemiology, School of Public Health and Department of Statistics, University of California, Los Angeles, USA

## Abstract

**Background:**

Prior research has shown a higher prevalence of substance use and mental disorders among sexual minorities, however, the influence of sexual orientation on treatment seeking has not been widely studied. We use a model of help-seeking for vulnerable populations to investigate factors related to treatment for alcohol or drug use disorders and mental health disorders, focusing on the contributions of gender, sexual orientation, and need.

**Methods:**

Survey data were obtained from a population-based probability sample of California residents that oversampled for sexual minorities. Logistic regression was used to model the enabling, predisposing, and need-related factors associated with past-year mental health or substance abuse treatment utilization among adults aged 18–64 (*N *= 2,074).

**Results:**

Compared with individuals without a diagnosed disorder, those with any disorder were more likely to receive treatment. After controlling for both presence of disorder and other factors, lesbians and bisexual women were most likely to receive treatment and heterosexual men were the least likely. Moreover, a considerable proportion of sexual orientation minorities without any diagnosable disorder, particularly lesbians and bisexual women, also reported receiving treatment.

**Conclusion:**

The study highlights the need to better understand the factors beyond meeting diagnostic criteria that underlie treatment utilization among sexual minorities. Future research should also aim to ascertain the effects of treatment provided to sexual minorities with and without diagnosable disorders, including the possibility that the provision of such treatment may reduce the likelihood of their progression to greater severity of distress, disorders, or impairments in functioning.

## Background

It is generally understood that the great majority of individuals with psychiatric disorders, including both mental and substance use disorders, do not receive treatment for them [1-3]. Many studies focusing on issues that pertain to unmet need for mental health treatment have found that underutilization of treatment is highest among those groups that are traditionally underserved, including the elderly, racial/ethnic minorities, those with low-incomes, the uninsured, and residents of rural areas [3,4]. It is also well documented that utilization of substance abuse treatment services is higher among individuals who have co-occurring mental disorders [5]. Further, treatment use varies by several key sociodemographic characteristics. For example, after controlling for number of disorders and other demographic characteristics, men with at least one past-year disorder had nearly twice the odds of having received substance abuse services, compared with women. In contrast, women were more likely to seek mental health treatment, after controlling for both the presence of psychiatric disorder and its severity [6]. One group that has been identified as heavier users of mental health services is lesbians, gay men, and bisexual individuals [7], although the reasons for this are not well understood [8]. This paper examines the relationship of gender and sexual orientation with treatment received for substance use or mental disorders in a population-based survey.

### Prevalence of substance use and mental disorders among sexual minority groups

Prior epidemiological surveys, both population-based and respondent-driven, have shown that minority sexual orientation populations report higher rates of drug use and related problems than do others [9,10]. Findings regarding alcohol use among sexual minorities are less consistent and often limited by the challenges of obtaining representative samples [11]. Analyses conducted with national survey data have shown lower rates of alcohol abstention and higher rates of alcohol use and problem drinking among homosexually active women compared with heterosexually active women, but no difference between homosexually active and heterosexually active men, controlling for sociodemographic characteristics [12]. In contrast, Hughes and colleagues found no differences between lesbians and heterosexual women in self-reported alcohol problems using national survey data [13]. Other studies have found few differences in alcohol consumption or symptoms of alcohol dependence among men with same-sex partners compared to men with opposite-sex partners [14,15]. However, there may be differences between gay men and lesbians in their patterns of substance use, with gay men having higher rates of inhalant and marijuana use compared with lesbians, and with older age associated with reduced marijuana use among lesbians, but not gay men [16].

Additional evidence comes from a population-based survey of women aged 18 to 29 in low-income neighborhoods in Northern California; women who reported having both male and female sexual partners had significantly higher rates of injection drug use compared with others [17]. Similarly, a survey of women in California showed that homosexually experienced women, particularly those who had both male and female sexual partners, reported higher and riskier alcohol use compared with exclusively heterosexually experienced women [18]. Stall and colleagues [10] surveyed men in 4 major urban areas who reported having male sex partners and found they had elevated levels of alcohol-related problems and recreational drug use. Moreover, their substance use was associated in complex ways with adverse early life circumstances, social and sexual practices, current mental health status, and degree of connection to gay male culture.

Elevated rates of some common mental disorders among sexual orientation minorities have also been demonstrated [19]. Using the National Comorbidity Survey, Gilman and colleagues found that women with same-sex sexual partners had a significantly higher likelihood of having any psychiatric disorder in the past year, including major depression, simple phobia, and posttraumatic stress disorder, compared with women who had only male partners [15]. Men reporting same-sex sexual partners were more likely than men reporting only opposite-sex partners to have an anxiety, mood, or substance use disorder. Cochran and colleagues [7] used national survey data to show that gay/bisexual men had a higher prevalence of depression, panic attacks, and psychological distress compared with heterosexual men, whereas lesbian/bisexual women had a greater prevalence of generalized anxiety disorder than heterosexual women. Last, a recent study showed higher rates of hazardous drinking, lifetime and current depression, and childhood sexual abuse among sexual minority women, compared with heterosexual women who were matched on demographics [20].

Several explanations have been posited for the generally higher prevalence of both substance use and mental health disorders among sexual minority populations. One study using national survey data showed that women who reported same-sex sexual partners spent more time in bars and party settings, and that these women consumed more alcohol in these settings, compared with exclusively heterosexual women [21]. Although gay men spent more time in bars than bisexual and heterosexual men, rates of heavy drinking among men did not vary by sexual orientation across settings. Thus, for lesbians especially, the social context of bars and parties may promote increased alcohol consumption [22].

Others studies have documented a link between having a sexual orientation minority status and exposure to life stressors, often stemming from experiences of discrimination and stigma [23], antigay violence or harassment (among men) [24], relative lack of coping skills [22], childhood adversity and familial rejection [25], and lack of other resources [26]. Indeed, the developmental challenges encountered by young gay/bisexual male youth often includes gay-related harassment and homophobic attacks, which have been associated with adverse health problems among adult gay men [27]. Moreover, several studies have demonstrated higher rates of psychological distress among gay, lesbian, or bisexual men and women, or homosexually experienced heterosexuals, as compared with individuals who were exclusively heterosexual, after adjusting for other confounding factors [7,24,28]. According to the "stress and vulnerability" model [29] and the "minority stress" model [30], these disparities in health among sexual minorities may be attributed to their cumulative exposure to harassment, maltreatment, discrimination, and victimization stemming from a hostile and homophobic culture. Thus, mental health and substance use disorders are not intrinsic to sexual minority orientation, but most likely result from the greater exposure to stressors typically experienced by sexual minorities, coupled with other individual and environmental risk factors [7,31,32]

### Treatment utilization among sexual minorities

Findings suggest that patterns of mental health and substance misuse treatment utilization among sexual minority groups differ from those of heterosexuals. For example, in a study using the 2000 National Alcohol Survey, Drabble and colleagues found that although lesbian and bisexual women had lower abstention rates overall, they were also more likely to report alcohol-related problems (e.g., being in fights or arguments, having conflicts with spouse/partner, losing time at work) and to have sought help for an alcohol problem [14]. Hughes and colleagues found that lesbians were more likely to report being in recovery or having received treatment for alcohol-related problems, although they consumed less alcohol than a matched sample of heterosexual women [13,33]. In a survey of over 2,000 lesbians and bisexual women recruited through multiple outreach strategies in California, only about two-fifths (41.5%) of respondents who reported impairment related to drug use had received lifetime professional help for a substance use problem and 16% wanted, but had not received, such assistance [34]. In another study using a population-based sample of women in Los Angeles County, Diamant and colleagues [35] found that lesbians and bisexual women were more likely than heterosexual women to use tobacco and alcohol, and, among lesbians, to drink heavily, however, they were less likely than heterosexual women to have health insurance, more likely to have been uninsured for health care in the preceding year, and more likely to have had problems in obtaining needed medical services.

In contrast, studies of mental health services utilization have shown that lesbians tend to utilize mental health services at higher rates and for longer duration as compared with heterosexual women [36]. One study showed that prior traumatic events, including childhood sexual abuse and physical abuse, were strongly associated with use of mental health services for lesbians, but were unrelated to treatment use for heterosexual women [37]. Another study used national survey data to examine receipt of mental health/substance abuse services among both men and women, comparing those with same-gender sex partners and those who were exclusively heterosexual [38]. Both men and women who had same-gender sex partners in the past year were more likely than their respective counterparts to have sought mental health/substance abuse services over the same period.

Taken collectively, these findings suggest that help seeking for mental health and substance abuse problems may be differentially influenced by sexual orientation status and gender. However, much of this work has been hampered by small sample sizes and limited assessment of clinical disorders. Further, sexual minorities who are also ethnic minorities face additional barriers to seeking health services and are less likely to receive care [39].

### Current paper

The goal of the present paper is to examine the relationship of gender and sexual orientation with treatment utilization for psychiatric problems, including both mental health (MH) and alcohol and drug (AOD) disorders. We apply the Gelberg-Andersen Behavioral Model for Vulnerable Populations [40]. This model, a modified version of the original Andersen behavioral model of health services utilization [41,42], posits a set of factors that influence services use. These include predisposing characteristics that exist prior to the perception of illness (e.g., race, education, age), resources that facilitate or, when lacking, impede health services utilization (e.g., income, health insurance, social support), and need variables that pertain to the type and severity of disorder(s). In addition to these domains, the expanded model for vulnerable populations takes into consideration other factors that may facilitate or impede services utilization among populations that encounter greater risks, such as residential instability, exposure to trauma and victimization, substance abuse and mental illness, and associated life stressors [43]. We hypothesize that individuals with sexual minority orientations will be more likely than heterosexuals to participate in treatment due to higher levels of stress and vulnerability, after controlling for predisposing, enabling, and need-related variables. We also anticipate the highest rates of treatment use to be among lesbians and bisexual women, reflecting the dual stress of vulnerability from both minority sexual orientation and gender.

## Methods

### Study design

Data for this study come from the *California Quality of Life Survey *(CalQOL), which is a follow-back to the 2003 *California Health Interview Survey *(CHIS). The parent CHIS is a stratified multistage random-digit telephone health surveillance interview of more than 42,000 adults aged 18 years and older that has been conducted every other year since 2001. Information collected covers a wide range of topics, including health status, health conditions, health-related behaviors, health insurance coverage, access to and use of health care services, and the health and development of children and adolescents.

The CalQOL follow-back survey used a subsample of the original CHIS survey sample to obtain more detailed information about specific topics (in this case, regarding sexual orientation and associated experiences). The overall CHIS response rate was 34% (using the American Association for Public Opinion Research Response Rate 4 method), which is consistent with other recent random-digit telephone interviews. In the CHIS, all adult respondents aged 18 to 70 years were asked about the genders of their sexual partners during the past year, and those aged 18 and older (with no age limit) were asked about their sexual orientation identity. Seventy-six percent of respondents were willing to participate in additional health surveys. From the CHIS sample, 4165 individuals were selected by probability methods. Eligibility was determined by having completed either a CHIS interview in either English or Spanish; a willingness to be recontacted; and an over-selection for sexual orientation minority status. Of these, 2322 individuals were successfully interviewed between October 2004 and February 2005 (56% response rate using the American Association for Public Opinion Research Response Rate 1 method).

### Study sample

The current study used data from 2074 individuals who were aged 18 to 64 years at the time of the CalQOL interview. Individuals aged 65 and older were excluded due to the nature of the study question (i.e., treatment received for psychiatric disorders) because insurance coverage in the United States is nearly universal after age 65 and may thereby mitigate other factors related to treatment utilization. Overall, the weighted sample was approximately half male (48.5%) and half female (51.5%). The ethnic/racial distribution was 55.6% White, 29.8% Hispanic, 5.9% African American, 7.8% Asian/Pacific Islander, and less than 1% Native American. About half of the sample was between the ages of 30 and 50, with a mean age of 40.7 [SD = 12.4] years. There were no significant differences between men and women in mean age or race/ethnicity classification. A majority (72.4%) were currently employed, although a smaller proportion of women than men were employed (65% vs. 80%, p < .0001). Most of the sample (81.2%) had health insurance (includes both public and private insurers). Nearly two thirds of the sample (64%) had at least some college education, with a slightly higher proportion of men than women having completed a college degree or more (38% vs. 35%, p < .001). A majority of the sample (64.3%) were currently married or living with a partner, with no significant difference observed between men and women.

### Measures

#### Treatment received

Treatment received was assessed by a question asking whether the respondent had "received any treatment for emotional, mental health, alcohol or other drug problems" in the past 12 months. Overall, 29.3% of the sample reported having received treatment for these problems in the past year. Among those receiving any treatment, 2% reported an inpatient hospitalization for either AOD or MH problems, 37% reported outpatient MH treatment, 68% used a prescription medicine for MH problems, 5% had outpatient AOD treatment, 5% attended 12-step meetings for AOD problems, less than 1% were treated in residential rehabilitation programs for AOD problems, 27% received treatment for MH or AOD problems from a primary care provider, and 20% reported use of alternative therapies (e.g., homeopathy, acupuncture, herbal treatments, spiritual healers) (data not mutually exclusive).

#### Substance use and mental disorders

Current (past 12-month) substance use disorders, including alcohol or drug abuse or dependence, were assessed using modified *DSM-IV *criteria [44]. Among those with any AOD disorder (data not mutually exclusive), 6.9% met criteria for alcohol abuse, 68.1% for alcohol dependence, 2.9% for drug abuse, and 40.4% for drug dependence (most often marijuana).

Assessment of mental disorders was based on the CIDI-SF, which renders probable diagnoses for past-year prevalence of major depression, generalized anxiety disorder, and panic attacks using *DSM-III-R *criteria [45]. Previous studies have demonstrated that there is moderate agreement between the trained lay interviewer-administered CIDI-SF diagnoses and those obtained by face-to-face diagnostic clinical interviews [46,47]. We also included a screen for current symptoms of post-traumatic stress disorder (PTSD). Among those in the sample with any MH disorder, 62.8% met criteria for major depression, 36.3% for generalized anxiety disorder, 28.2% for panic attacks, and 26.1% screened positive for PTSD (data not mutually exclusive).

Respondents were categorized into one of four groups on the basis of evidence of any past-year AOD or MH disorder: no evidence of any disorder (71.8%), an alcohol or drug (AOD) disorder only (3.8%), a mental health (MH) disorder only (20.6%), and both an AOD and MH disorder (3.8%). Because of the small sample sizes for two of these categories, a dichotomous variable indicating any AOD and/or MH disorder was used in the multivariate analyses (described below).

#### Predisposing variables

These included age, race/ethnicity, and gender/sexual orientation. Sexual orientation was determined by information obtained from respondents about both their behavioral histories and self-identification. Behavioral questions asked about the genders of sexual partners since age 18 years and during the past year. Individuals were also asked their sexual orientation identity. Respondents were classified into one of two sexual orientation categories by gender: lesbian, gay, bisexual, or homosexually experienced women (12.3%; hereinafter referred to as "lesbian/bisexual women"); gay, bisexual, or homosexually experienced men (14.1%; hereinafter referred to as "gay/bisexual men"); exclusively heterosexual women (39.4%); and exclusively heterosexual men (34.2%).

#### Enabling characteristics

These items pertain to characteristics of individuals that have been identified in previous studies as facilitating access to health services, such as human capital, resources, and social support [40-42]. They included employment status (i.e., in the labor force or not); insurance status, which was assessed with several questions asking whether respondents had private or government-sponsored health coverage from various sources; level of educational attainment, scored with a 5-level ordinal variable ranging from *less than high school *to *post-college education; *and whether the individual was married or "living with a partner in a marriage-like relationship." In addition, we created a measure of *global social support *using items from the *MOS Social Support Scale *[48]. Respondents were asked about the amount of support they had received from others in the past 4 weeks in 6 areas (i.e., daily chores when sick, feeling loved/wanted, talk about problems, have a good time with others, give information, and give money). Each item was rated on a Likert scale ranging from 1 = none of the time to 5 = all of the time; items were summed into a total score (alpha reliability = 0.86), which was dichotomized as either *high *or *low*.

### Statistical analyses

Data were analyzed with SAS version 9.1.3. We applied sample weighting to adjust for selection probability, nonresponse, and post-stratification to generate estimates representative of the California population. Past-year treatment received was examined by gender/sexual orientation groups and by type of disorder using cross-tabulations. Any treatment received in the past year was modeled as a dependent variable and predisposing, enabling, need-related, and gender/sexual orientation factors were included simultaneously as independent variables in multiple logistic regression models. To determine the odds of help-seeking for each gender/sexual orientation group vis à vis the others, three identical models were tested in which the referent group was rotated, while all other variables were kept constant. Odds ratios (OR) and 95% confidence intervals (CI) are presented. Statistical significance is determined at the *p *< 0.05 level. This study received institutional review board approval from the University of California, Los Angeles.

## Results

### Treatment received by gender, sexual orientation, and disorder

Overall, there is a main effect of sexual orientation on treatment received; 48.5% of lesbian/gay/bisexual individuals reported receiving treatment in the past year as compared to 22.5% of heterosexuals (*χ*^2 ^[1] = 131.6, *p *< .0001). Similarly, there is a main effect of gender, with about one third of women (33.8%) and one quarter of men (24.5%) reporting receiving treatment in the past year (*χ*^2 ^[1] = 21.7, *p *< .0001). As would be expected, the rate of treatment received varied by disorder status, with 18.4% of those with no disorder, 37.2% of those with an AOD disorder only, 58.1% of those with a MH disorder only, and 73.4% of those with both types of disorders reporting having received some form of treatment in the past year (*χ*^2 ^[df = 3] = 331.5, *p *< .0001).

In Table 1 we show the distributions for treatment received categorized by sexual orientation and type of disorder for men and women separately. A greater proportion of gay/bisexual men than heterosexual men reported receiving treatment in the past year (42.5% vs. 17.1%); similarly, a greater proportion of lesbian/bisexual women than heterosexual women received treatment in the past year (55.3% vs. 27.1%).

**Table 1:** Mental health or substance abuse treatment received in past year by gender, sexual orientation, and disorder

		Women				Men			
			
	Total (N = 2074)	Heterosexual (*n *= 816)	LB (*n *= 255)	Total (*N *= 1071)	χ^2^ (*df *= 1)	Heterosexual (*n *= 709)	GB (*n *= 294)	Total (*N *= 1003)	χ^2^ (df = 1)
No disorder (*n *= 1490)	18.4	16.9	43.7	22.0	48.10***	9.5	30.7	14.9	49.80***
AOD disorder only (*n *= 78)	37.2	50.0	40.0	46.2	0.25	34.4	30.0	32.7	0.11
MH disorder only (*n *= 427)	58.1	55.1	71.6	60.6	6.67**	41.9	70.0	54.0	12.70***
Both AOD & MH disorders (*n *= 79)	73.4	71.4	80.0	75.0	0.34	67.9	80.0	72.1	0.72

Total (*N *= 2074)	29.4	27.1	55.3	33.8	68.80***	17.1	42.5	24.5	72.60***

Notes:N's are unweighted, statistics are weighted; LB = lesbian/bisexual; GB = gay/bisexual

**p < .01, ***p < .001

Among women without a disorder, a greater proportion of lesbian/bisexual women than heterosexual women indicated that they had received treatment (43.7% vs. 16.9%). Similarly, among women with a MH disorder only, a greater proportion of lesbian/bisexual women than heterosexual women had received treatment (71.6% vs. 55.1%). There were no significant differences in treatment received by sexual orientation among women with an AOD disorder only or with both an AOD and MH disorder.

Among men without a disorder, a greater proportion of gay/bisexual men than heterosexual men reported receiving treatment in the past year (30.7% vs. 9.5%). The same pattern was observed among men with a MH disorder only, with 70% of gay/bisexual men and 41.9% of heterosexual men having received treatment. There were no significant differences in treatment received by sexual orientation for men who had an AOD disorder only or had both an AOD and MH disorder.

### Logistic regression model of past-year treatment use

We next developed a logistic regression model testing the independent contributions of predisposing, enabling, and need-related factors on past-year treatment utilization in which we contrasted the reference group for gender/sexual orientation status. In Table 2 we show the findings from this model (pseudo R^2 ^= 0.279). Among the predisposing variables, older age increased the likelihood of receiving treatment (adjusted OR = 1.01). As compared with Whites, Hispanics (adjusted OR = 0.46), African Americans (adjusted OR = 0.34), and Asian/Pacific Islanders (adjusted OR = 0.29) were all less likely to have received treatment. Considering enabling characteristics, we observed no independent significant differences in treatment received by employment, insurance, education, level of social support, or marital/partner status.

**Table 2:** Logistic regression model predicting treatment received in past year (N = 2,074)

	Model 1		Model 2		Model 3	
Variable	OR	95% CI	OR	95% CI	OR	95% CI
**Predisposing Characteristics**						
Age	1.01	1.004, 1.02*		--		--
Ethnicity (ref = white)						
Hispanic	0.46	0.35, 0.65***		--		--
African American	0.34	0.19, 0.62***		--		--
Asian/Pacific Islander	0.29	0.17, 0.54***		--		--
Native American	1.20	0.31, 3.28		--		--
**Enabling Characteristics**						
Employed (Y/N)	0.78	0.61, 1.04		--		--
Insured (Y/N)	1.22	0.86, 1.68		--		--
Level of education (1–5)	1.00	0.90, 1.11		--		--
Social support (high/low)	0.92	0.71, 1.26		--		--
Married or partnered (Y/N)	0.85	0.66, 1.09		--		--
**Need Characteristic**						
Any AOD or MH disorder (vs. none)	6.23	4.90, 7.92***		--		--
**Gender/Sexual Orientation**						--
Female heterosexual	referent			--		--
Female lesbian/bisexual	2.08	1.25, 3.47**	referent			--
Male heterosexual	0.57	0.44, 0.73***	0.27	0.16, 0.46***	referent	
Male gay/bisexual	1.57	1.01, 2.45*	0.75	0.40, 1.41	2.76	1.76, 4.36***

Notes: OR = odds ratio, CI = confidence interval, AOD = alcohol or other drug, MH = mental health

*p < .05, **p < .01, ***p < .001

The presence of a MH and/or AOD disorder significantly increased the odds of receiving treatment (adjusted OR = 6.2). In another set of models (data not shown), when the separate categories for MH and/or AOD disorders were entered (with the referent group set to "no disorder"), there was a graded relationship between type of disorder and treatment received. Individuals with only an AOD disorder evidenced a greater odds (adjusted OR 4.6; 95% CI: 2.8, 7.8) of receiving treatment, as did those with only a MH disorder (adjusted OR = 5.6; 95% CI: 4.3, 7.3) and those with both an AOD and MH disorder (adjusted OR = 17, 95% CI: 9.7, 31.3) as compared to those with no disorder (all significant at *p *< .0001). However, given the small cell size of two of these categories (AOD only and combined AOD and MH disorders), these estimates are less stable.

With regard to gender and sexual orientation considered simultaneously (see Model 1), when heterosexual women are treated as the referent group, both lesbians and bisexual women (adjusted OR = 2.08) and gay and bisexual men (adjusted OR = 1.57) had greater odds of receiving treatment, but heterosexual men had about half the odds of heterosexual women (adjusted OR = 0.57). As shown in Model 2, in which lesbians and bisexual women were treated as the referent group, both heterosexual women (adjusted OR = 0.48) and heterosexual men (adjusted OR = 0.27) were less likely to report having received treatment. But there was no significant difference in the odds of treatment received between lesbians/bisexual women and gay/bisexual men. In Model 3, when heterosexual men were classified as the referent group, all other groups were significantly more likely to receive treatment (heterosexual women [adjusted OR = 1.76], gay and bisexual men [adjusted OR = 2.76], and lesbians and bisexual women [adjusted OR = 3.66]).

## Discussion

This study builds upon previous epidemiological studies that have shown higher prevalences of AOD and MH disorders among sexual minority populations [7,8,11,13,15]; we extend these findings by showing that treatment utilization for these disorders varies by both gender and sexual orientation. The study findings are strengthened by the use of a population-based sample and a theoretically guided model of health services utilization. In the broader literature it is well known that health services utilization is greater among women generally. Here we have shown that minority sexual orientation is also an important explanatory variable in understanding treatment seeking among women. Lesbians and bisexual women appear to be approximately twice as likely as heterosexual women to report having received recent treatment for mental health or substance use disorders, after controlling for the presence of either type of disorder and other predisposing and enabling characteristics. Indeed, more than half of the lesbians and bisexual women in the study indicated that they had received services in the past year for mental health or substance use-related problems. Further, this sexual-orientation-related effect was also seen among gay and bisexual men who were significantly more likely than both heterosexual men and women to report having received recent treatment, after controlling for other factors.

The greater propensity for treatment use among those possessing a minority sexual orientation may be related to several factors. These include differential norms that promote help-seeking among sexual minorities in general, particularly among lesbians and bisexual women, as well as higher exposure to discrimination, violence, and other stressful life events [8,23,30,31,49-52]. Further, the pervasive and historically rooted societal pathologizing of homosexuality [53-57], particularly among racial/ethnic minorities by their communities, may contribute to this propensity for treatment by construing homosexuality and issues associated with it as mental health problems. This cultural definition may indirectly function as a predisposing factor that encourages the seeking of professional help for problems that are assumed to derive from individual distress, or from the internalization of the stigma ascribed to homosexuality by some [58]. Further, the culture of gay and lesbian communities may increase the social norms and expectations that therapeutic services are appropriate places for coping with the stresses associated with being a sexual minority.

As anticipated, rates of receiving treatment varied by severity of the disorders that occurred during the period of interest. It is reassuring, for example, that nearly three quarters of individuals meeting criteria for both substance use and mental health disorders indicated that they had received at least some services in the past year. At the same time, nearly 20% of individuals who did not have a diagnosable disorder in the past year reported having received some form of mental health and/or substance abuse-related services. This finding is consistent with national surveys showing that many individuals who receive mental health treatment do not have a diagnosable disorder [60], but may have other symptoms, such as psychological distress or impairments in functioning, that lead them to seek care [60-62]. Moreover, these findings have called into question the criteria that should be used to indicate "need for treatment," apart from diagnostic criteria, as well as the basis for determining the adequacy of the treatment system in providing treatment to those who feel they need it (including those with and without diagnosed disorders) [63]. This is a particularly salient issue for understanding treatment utilization among sexual orientation minorities, many of whom in this study sought services in the absence of evidence of either a mental health or substance use disorder. Why this is so is unclear but suggests either an over-utilization of care or that estimates of unmet need in this population are less dependent on the presence of diagnosed disorders. Moreover, this finding has implication for estimating need for health services, which is typically based on prevalence estimates of disorders.

None of the enabling characteristics that have been associated with treatment seeking in other studies (i.e., employment, insurance, education, social support, and marital/partner status) were significantly related to treatment use in the multivariate models. It is possible that the effects of disorder and sexual orientation cancelled out any effects associated with these factors. However, we observed that ethnic/racial minorities were less likely to utilize mental health or substance use related services. This effect was found after controlling for differences in morbidity and other predisposing and enabling characteristics, including health insurance, which have been associated with underutilization of these services among ethnic minorities in prior research [64-66]. African Americans and Hispanics may underutilize services for mental health and substance use problems for a variety of reasons, including a lack of familiarity with the types of services available [67]; prior negative experiences with service providers [68]; or because of greater stigma attached to use of these services by their families and communities [69,70]. Further, there are differences among women in utilization of these services by both race/ethnicity and sexual orientation [71,72]. Exploration of the interactions among gender, sexual orientation, and race/ethnicity on treatment use is beyond the scope of the present paper, but is an area in need of more investigation.

### Study limitations

This study encountered several limitations typical of telephone-based follow-back surveys. The California Quality of Life Survey sample was recruited by recontacting those 2003 CHIS respondents who had agreed to be recontacted, using the telephone number associated with the original interview. Loss to follow-up was most often due to mobility from the original residence and was associated with younger age. Thus our estimates of the relationship between age and treatment received may be imprecise; other factors associated with lack of contact for the follow-up survey may also have influenced the estimates derived from the study sample. Although the follow-back survey oversampled for sexual minorities, the cell sizes for groups defined by sexual orientation and type of disorder (particularly among those with an AOD disorder only or with both MH and AOD disorders) were small (approximately 78 cases). Hence, statistical power was somewhat limited and may have failed to detect some relationships among sexual orientation, type of disorder, and treatment received. Lastly, although the study findings may be generalized to the general population in California, the dependent variable of interest, treatment seeking, may be particularly influenced by the cultural context of California, in which therapeutic interventions are consistent with an overall "therapy culture" [73], thus limiting generalizability to other locations that differ in this regard.

## Conclusion

The study provides important evidence of the differential effects of gender and sexual orientation minority status on the receipt of mental health and substance abuse treatment, beyond the influence of the presence of a diagnosable disorder and other factors that predispose individuals to seek treatment. The findings showed that minority sexual orientation predisposes individuals to seek out services, despite pervasive barriers that exist within the service delivery system that might even discourage their use by this population [74]. The study findings have implications for allocation of public funding for the provision of public mental health and substance abuse treatment. When projecting the treatment needs of sexual orientation minorities, service planning should take into consideration the effects of environmental and life stressors, including experiences of discrimination, violence, and hate crimes. Moreover, these findings suggest important areas for future investigation regarding the receipt of treatment for mental health or substance use disorders, including the influence of psychological distress, impairments in functioning, and social norms that support or hinder treatment seeking, and how these factors operate differentially for men and women of varying sexual orientations. Further, research is also needed to ascertain the effects of treatment provided to individuals who do not have diagnosable disorders, including the possibility that the provision of such treatment may reduce the likelihood of their progression to greater severity of distress, disorders, or impairments in functioning. Last, a better understanding of the factors that encourage treatment seeking among sexual orientation minorities, especially lesbians and gay women, may generate knowledge that can be used to improve delivery of treatment to those who would benefit from it or who currently underutilize treatment.

## Competing interests

The authors declare that they have no competing interests.

## Authors' contributions

CEG conceived the idea for the paper, directed the data analyses, and drafted the paper; LG conducted the statistical analyses and contributed to the interpretation of findings and writing of the paper; VMM collaborated on the design of the original survey study and contributed to the interpretation of findings and writing of the paper; SDC conceived and directed the original survey study and contributed to the interpretation of findings and writing of the paper. All authors read and approved the final manuscript.

## Pre-publication history

The pre-publication history for this paper can be accessed here:


